# Pancreatic Perfusion CT in Early Stage of Severe Acute Pancreatitis

**DOI:** 10.1155/2012/497386

**Published:** 2012-02-15

**Authors:** Yoshihisa Tsuji, Naoki Takahashi, Chiba Tsutomu

**Affiliations:** ^1^Department of Gastroenterology and Hepatology, Kyoto University Graduate School of Medicine, Shogoin, Sakyo-ku, Kyoto 606-8507, Japan; ^2^Division of Radiology Mayo Clinic, Rochester MN 55905, USA

## Abstract

Early intensive care for severe acute pancreatitis is essential for improving SAP mortality rates. However, intensive therapies for SAP are often delayed because there is no ideal way to accurately evaluate severity in the early stages. Currently, perfusion CT has been shown useful to predict prognosis of SAP in the early stage. In this presented paper, we would like to review the clinical usefulness and limitations of perfusion CT for evaluation of local and systemic complications in early stage of SAP.

## 1. Introduction

**Figure 1:**
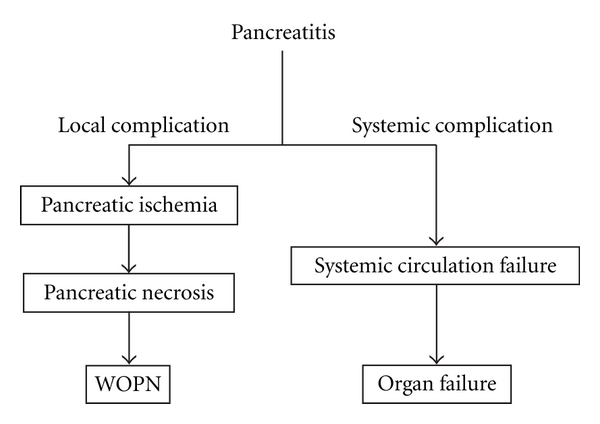
Schema of complications of severe acute pancreatitis. WOPN: walled-off pancreatic necrosis.

Severe acute pancreatitis (SAP) is a fatal disease [1]. The Atlanta Symposium criteria for the severity of acute pancreatitis define SAP as acute pancreatitis with local complications (pancreatic necrosis, abscess, and pseudocysts) and/or with systemic complications (organ failure, disseminated intravascular coagulation, and severe metabolic disturbances) [2] (Figure 1). Both acute necrotizing pancreatitis (ANP) and multiple-organ failure (MOF) have been shown to be significant prognostic factors [3–6]. Mortality rates for SAP patients developing ANP and MOF are 32% and 30%, respectively [7]. Early intensive care for SAP is essential for improving SAP mortality rates [8–10]. However, intensive therapies for SAP are often delayed because there is no ideal way to accurately evaluate severity in the early stages [11–13].

Perfusion CT has been used for evaluation of various pancreatic diseases [14–22]. Perfusion CT has been shown useful to predict prognosis of SAP in the early stage [17, 18]. In this presented paper, we would like to review the clinical usefulness and limitations of perfusion CT for evaluation of local and systemic complications in early stage of SAP.

## 2. Perfusion CT Technique

Previously published perfusion CT protocols are summarized in Table 1. Multidetector CT (MDCT) is essential for performing perfusion CT of pancreas. With a 4–64 slice MDCT scanner, perfusion CT scans are obtained with the patient in a stationary position. The craniocaudal CT scan coverage is limited to 20 to 32 mm (4 slices of 5 to 8 mm thickness). Therefore, scan location must be carefully chosen to cover as much of the pancreas as possible as it is often difficult to cover the entire pancreas. Since most pancreatic necrosis occurs in the neck region, it is probably uncommon to exclude the area of necrosis due to the scanning coverage limitation. With the use of a recently developed 256–320-slice MDCT scanners [19], craniocaudal coverage has increased to 80–160 mm. Alternative way to increase the craniocaudal coverage is by using the so-called shuttle or toggle mode. In this mode, similar to conventional CT scans, patient table moves back and forth as the multiple scans are performed.

First, noncontrast transaxial images of the upper abdomen are obtained using low-dose technique. This scan is performed to localize the pancreas, and it determines the scan range of the perfusion CT.

Perfusion CT is performed after a bolus injection of intravenous contrast material. Unlike conventional CT, the perfusion CT requires smaller dose (40–50 mL) of contrast material injected at a high rate (4–10 mL/sec). Higher concentration of contrast material (350–370 mgI/kg) is preferred [23–25].

Perfusion CT images are obtained multiple times through the pancreas. In most of previous reports, scan interval ranges from 0.5 to 1.5 second, and the scan duration ranges from 30 to 150 seconds, respectively (Table 1). Total scan duration necessary for calculation of perfusion parameters may depend on the algorithm used. For example, maximum slope method needs shorter duration scan time than the deconvolution method [14, 19, 26]. Because the scan duration is long, the scans are usually performed under free breathing.

Perfusion CT scan is obtained at a low tube current (mAs) to reduce radiation dose. At 120 kV, mAs of 100 is commonly used. There is increased interest in the use of low tube voltage setting, as it reduces radiation dose and improves iodine contrast material conspicuity. In a smaller patient, the use of 100 kV or 80 kV is recommended. In a larger patient, the use of low-kV scan may result in noisy images due to photon deficiency.

### 2.1. Radiation Dose and Scan Parameters

Radiation dose is dependent on the tube current (mAs), tube voltage (kV), number of scans, and scan coverage [29]. Tube current (mAs) and tube voltage (kV) are largely dictated by the patient size to maintain adequate image quality. Radiation dose should be kept as low as reasonably achievable (ALARA) by reducing the scanning parameter settings but achieving image dataset adequate for calculating CT perfusion parameters [27, 30]. Further study is necessary to optimize the scanning protocol.

From a European study, the effective dose of pancreatic perfusion CT was 3.54 mSv with 90 KV, 100 mAs, and 40 scans [17]. A study from Japan reported that mean radiation dose of pancreatic perfusion CT was approximately 204.8 mGy**·**cm (dose-length product (DLPw)), 3.07 mSv (effective dose), and 64 mGy (CT dose index volume (CTDIvol)) with 80 kV, 60 mAs, and 106 scans [27]. In the national survey, the radiation exposure of a single-phase abdominal CT was 13–25 mGy (CTDIvol) [31]. Therefore, the radiation dose of perfusion CT is slightly higher than that of biphasic (two phase), which is commonly used for pancreatic or liver imaging. Average abdominal transverse diameter of the Japanese patients in our experience was 32 cm, while transverse diameters of patients in the Unites States are usually larger [32]. Therefore, the radiation dose will likely be higher in the western countries

### 2.2. Perfusion CT Data Analysis

Pancreatic perfusion CT image data are analyzed by using perfusion CT analysis software. There are various perfusion algorithms to calculate perfusion parameters. Maximum slope method, deconvolution method, single-compartment method, and the Patlak method are commonly used perfusion algorithms. Which perfusion best suits in the evaluation of SAP is yet to be determined. As different perfusion algorithms are suited for different disease processes and require different scanning protocol, determination of scanning protocol and perfusion algorithms should be considered together. For example, maximum slope method may require shorter scanning duration, but higher rate of contrast injection is required, while deconvolution method may require longer scanning duration but slower rate of contrast injection rate [14, 26].

The software requires placement of small regions of interest (ROI) on an artery to generate arterial input function. Venous outflow function is required in deconvolution method. This process is required because the computer algorithm compares the shape and height of the time-density curve of each pixel of the pancreatic CT time series with shape and height of the arterial and/or venous time-density curves to calculate pancreatic perfusion parameters. Calculated pancreatic perfusion parameters are displayed using color maps [14].

## 3. Perfusion CT for Predicting Development of Pancreatic Necrosis in the Early Stage of Severe Acute Pancreatitis

Development of pancreatic necrosis is the critical event of acute pancreatitis that determines the prognosis of the patients. The overall mortality rate of acute pancreatitis is reported to be between 2.1% and 9.2% worldwide [1]. Pancreatic necrosis occurs in 10–15% of patients with SAP, with a mortality rate of 23% [1]. This rate is nearly twice that for patients with SAP who do not develop pancreatic necrosis (i.e., 11%) [1].

There is a report that dynamic contrast-enhanced CT is more accurate than either the Ranson criteria for pancreatitis mortality or the APACHE II scoring system in predicting the development of pancreatic necrosis [33]. However, the accuracy of contrast-enhanced CT in predicting necrosis at an early stage of SAP is not satisfactory [34]. The United Kingdom guidelines for the management of acute pancreatitis, the most popular clinical guideline of acute pancreatitis, recommends that contrast-enhanced CT should be performed at day 3 or later after onset of SAP because of its low sensitivity of CT [11].

In our experience, perfusion CT performed within 3 days of onset of symptoms had a sensitivity and specificity of 100% and 95.3% for predicting development of pancreatic necrosis [18]. The area of necrosis was depicted as area of pancreatic blood flow decreased by more than 19.3% of surrounding pancreatic parenchyma. The area of perfusion defect was commonly diagnosed by using pancreatic blood flow. The perfusion defects detected by perfusion CT reflected ischemia which was produced by vasospasms of the intrapancreatic arteries [35, 36].

## 4. Perfusion CT for Evaluating Systemic Blood Flow

Perfusion CT could be a useful tool to evaluate abnormal systemic circulation in early stage of SAP. Recent study by Whitcomb et al. showed that elevated serum angiopoietin-2 (Ang-2) on admission is predictive of persistent organ failure in patients with sap [37]. Ang-2 is produced by damaged vessels and increases vascular permeability [38]. In our study, elevated serum Ang-2 is related with hyperdynamic state of systemic circulation [22]. In this study, perfusion CT parameter (*τ*) was calculated using single-compartment model [28, 39]. *τ* is a measure of the mean transit time of contrast material from upper abdominal aorta to pancreas; thus, this could be considered a surrogate of systemic circulation with a lower value indicating hyperdynamic state of systemic circulation [28]. In the result, significant correlation was found between *τ* and serum Ang-2 levels (**P** < 0.05); higher serum Ang-2 levels were associated with lower *τ* values (hyperdynamic state of systemic circulation).

Hepatic circulation abnormality has been reported in patients with SAP using Perfusion CT [40]. They reported that hepatic arterial perfusion is increased in the early stage of SAP as measured on dual-input maximum slope method.

## 5. Clinical Utility of Pancreatic Perfusion CT

Early diagnosis of pancreatic necrosis is very important in the treatment of patients with SAP. Current methods to predict early pancreatic necrosis or SAP is not satisfactory [11–13]. Perfusion CT is a promising technique that allows accurate diagnosis of pancreatic necrosis. Early diagnosis allows prompt clinical decision such as transferring patients to ICU or institution of aggressive treatment such as anticoagulation therapy [41], continuous regional arterial infusion of antiprothrombin agent [8, 9], early fluid resuscitation [10], and molecular targeted therapy [42, 43].

## 6. Conclusion

Perfusion CT is a promising technique for diagnosis of local and systemic complications of SAP at an early stage.

## Figures and Tables

**Table 1:** Scanning protocols of pancreatic perfusion CT.

Authors	Disease	CT	The number of detector	kv	mA	Images	Contrast matter		Duration time (sec)	Algorithm
							Injection rate (mL/sec)	Amount		
Miles [25]	—	—	—	—	50–100	60	4–7	40 mL	60	Deconvolution
					100–250	15	7–10	50 mL	45	Compartment
					100–250	6	4	100 mL	120	Patlak plot
Tsushima and kusano [15]	Normal	S	1	—	—	19	5	40 mL	85	Maximum slope
Abe et al. [16]	PC	G	1	120	60	—	5	0.5 mL/kg	40	Deconvolution
Bize et al. [17]	AP	P	16	90	100	40	5	40 mL	40	Maximum slope
Tsuji et al. [18]	AP	T	16/64	120	30–50	30–48	4	40 mL	33–48	Deconvolution
Tsuji et al. [27]	AP/NET	T	64	80	40	106	4	40 mL	54	Deconvolution
Sheiman and Stick [28]	Normal	G	64	100	80	30	4	40 mL	90	Compartment
d'Assignies et al. [20]	NET	G	64	100	100	70	4	40 mL	150	Compartment
Park et al. [21]	PC	S	64	100	100	30	5	50 mL	30	Patlak plot
Kandel et al. [19]	PC	T	320	100	45	19	8	60 mL	80	Maximum slope

PC: pancreatic cancer; AP: acute pancreatitis; NET: neuroendocrine tumor; S: Siemens; G: GE Health care; P: Philipse; T: Toshiba.
